# Parametric study for optimizing double-layer microchannel heat sink for solar panel thermal management

**DOI:** 10.1038/s41598-022-23061-8

**Published:** 2022-10-31

**Authors:** Hesham I. Elqady, A. H. El-Shazly, M. F. Elkady

**Affiliations:** 1 Chemical and Petrochemicals Engineering Department, Egypt-Japan University of Science and Technology, Alexandria, 21934 Egypt; 2 Faculty of Engineering, Aswan University, Aswan, 81528 Egypt; 3 Chemical Engineering Department, Faculty of Engineering, Alexandria University, Alexandria, 11432 Egypt; 4 Fabrication Technology Department, Advanced Technology and New Materials Research Institute (ATNMRI), City of Scientific Research and Technological Applications, Alexandria, Egypt

**Keywords:** Solar energy, Solar thermal energy

## Abstract

The most significant issue affecting the electric efficiency of solar panels is overheating. Concentration photovoltaic (CPV) modules work by converting approximately 80% of sunlight to heat; this may exceed the cell operating temperature limits. Therefore, thermal management is the best choice for keeping such panels working under specified conditions. Prior to producing an actual solar indoor unit, the current research primarily focuses on optimizing the heat sink dimensions that affect the cooling performance of the solar panel. Two parametric studies were employed to optimize the microchannel heat sink design. First, a two-dimensional numerical study was implemented to optimize the best channel height for more uniform flow inside a double-layer microchannel heat sink (DL-MCHS); the width of channels was kept as a constant value. Second, a three-dimensional conjugate heat transfer model for fluid flow in the optimized heat sink was used to optimize the inlet/outlet header length. To evaluate the overall CPV performance, a further numerical case study was carried out for the optimized designs at a wide range of inlet mass flow rates and steady-state heat flux. The findings indicated that a channel height of 0.5 mm and a header length of 20 mm were the best design points for the suggested heat sink. To assess the effectiveness of a solar/thermal module, the selected design points were applied to a 3D model. The maximum electricity efficiency measured was 17.45%, nearly 40% greater than the typical CPV/T system.

## Introduction

One of the main objectives of adopting new technology is to downsize electronic and semiconductor devices. The problem might hinder the development of ultra-dense circuitry since greater heat causes system performance to break down. By 2026, computer chips' expected highest heat flux will exceed 4000 W/cm^2^^1^. Therefore, the generated average temperature through devices' surfaces will harm electronic equipment operation. Concentrated photovoltaic (CPV) modules, also known as high-heat-flux systems, are a type of semiconductor applications device that is extremely temperature-sensitive^2^. The massive solar radiation flux may cause CPV layers difficulties, such as physical damage and alternative thermal expansion. Therefore, the system's overall performance and lifetime will decrease^3^. Thermal management has become an important solution for keeping solar panels working under specified operating conditions; liquid cooling is a powerful and innovative method of thermal management^4^.

Multiple research investigations have emphasized the essential benefits and drawbacks of using active cooling as the principal mode for operating solar panels within limited conditions^2,5^. Optimizing MCHS geometrical factors that substantially influence local heat transfer is strongly recommended. Naqiuddin et al.^6^ reported that thermal performance is influenced by two major factors: channel type and aspect ratio. Their results showed a suitable improvement in thermal performance at a lower aspect ratio. Gong et al.^7^ conducted a detailed numerical analysis to optimize the thermal efficiency of four heat sink types. They reported that the double-layer microchannel heat sink (DL-MCHS) outperformed the rectangular column fin and single-hole jet-cooling heat sinks in terms of cooling capabilities. Alfellag et al.^8^ developed numerical optimization research for determining the optimal fin angle inside the cooling device. The investigated heat sink's total fluid performance improved when the slots' inclination angle was raised to 55 degrees.

Further studies have been conducted to optimize the design of the microchannel heatsink device for high-flux applications^9,10^. For Example, Wang et al.^11^ used a multi-objective evolutionary algorithm to optimize the double-layered MCHS with semi-porous ribs. It demonstrates that cooling performance is enhanced by 14.06% compared to the original design, and pumping power is lowered by 16.40%. A compromise must be made to get the best performance between the upper channel's pumping capacity and the lower channel's cooling capacity. Leng et al.^12^ investigated several factors to achieve the best heat sink performance, including channel number, channel width, bottom channel height, and bottom coolant inlet velocity.

Based on the authors' literature survey, optimizing the geometrical characteristics of cooling devices strongly impacts local heat transfer. It is strongly advised to include the effect of headers in the numerical consideration to predict the solar system's actual hydraulic and thermal performance^13^. Therefore, the ongoing research work is divided into three main parts. First, an optimization of the divergent microchannel is employed to enhance the capability of using the suggested heat sink as an effective cooling device for solar cells. To achieve this, a multi-objective optimization procedure is utilized using a two-dimensional model to optimize the height for each divergent channel; the width was kept constant. Second, a three-dimensional conjugate heat transfer model for fluid flow in the optimized heat sink was used to optimize the inlet/outlet header length. The main key factor is to achieve a uniform flow through channels and prevent maldistribution in the inlet ports. Finally, the selected design of the DL-MCHS is numerically attached with the suggested cell by the authors' earlier work at a wide range of inlet mass flow rates and 5 suns concentration ratio (CR)^14^. The created study was utilized using a 3D heat transfer model. Under laminar flow conditions, water was chosen as the main coolant and temperature-dependent thermophysical properties^15^.

### Physical model description

**Figure 1:**
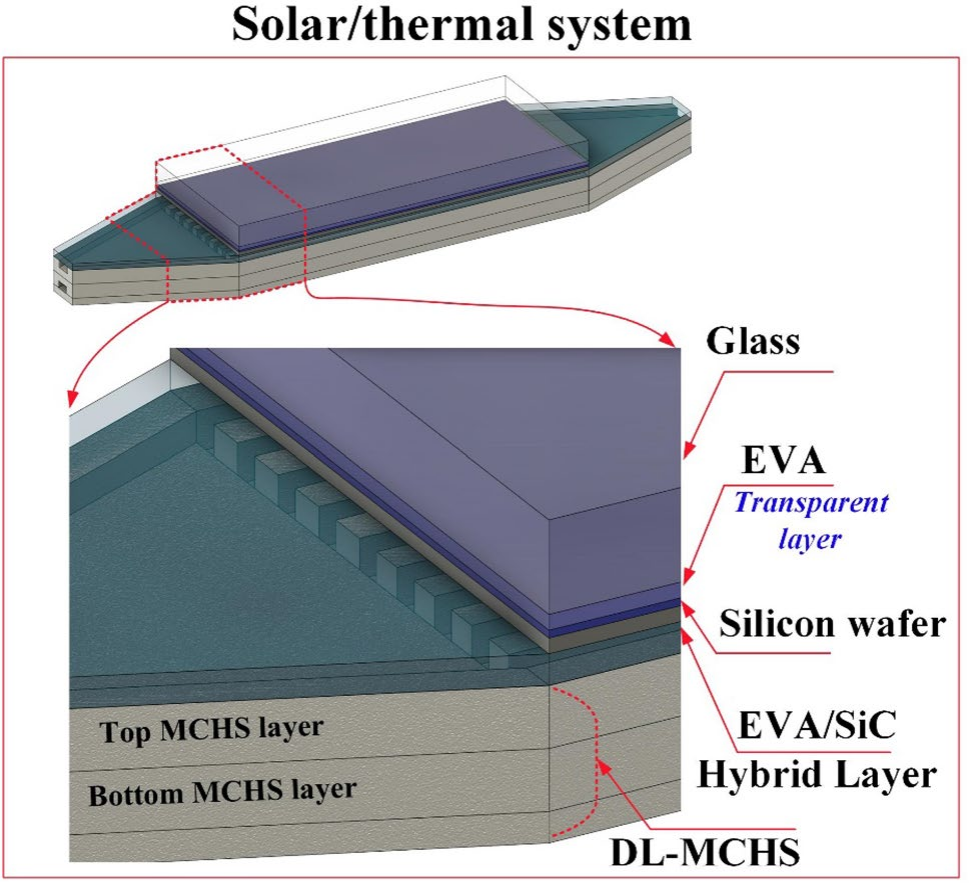
Schematic diagram for a three-dimensional concentrated photovoltaic module includes a double-layer microchannel heat sink device in the backside.

As shown in Fig. 1, the CPV system employed in the investigation is composed of two important parts, a solar panel and a heat sink. The solar panel contains four layers: the glass cover layer, the silicon wafer layer, and two encapsulant polymeric materials layers. The upper encapsulant layer is made from ethylene–vinyl acetate (EVA), while the lower from EVA and silicon carbide (SiC). This system is also suggested based on the authors' earlier study^14^. The heat sink part, consists of two identical layers, one above the other. Each layer contains 12 identical microchannels. This configuration is an effective coolant device for keeping the solar panel module operating under the recommended condition. To prevent the solar cell from overheating, the DL-MCHS is attached directly to the backside of the solar cell. The uniform distribution of coolant inside channels plays a vital role in generating uniform temperature through the PV surface^16^. Therefore, counter flow (CF) operation is considered the best flow direction for achieving such uniformity^14^.

### Theoretical analysis

#### Optimization procedures

The simulation of the current study is divided into two parts. The first is for microchannel height optimization, while the second is for header length. To optimize heatsink dimensions, only geometry variables were considered during the simulation. The lengths of the headers and the height of the microchannel serve as design variables during simulation. The numerical solution was performed using ANSYS FLUENT design exploration software with Dell Precision 5820 workstation Intel Xeon® processor of 3.75 GHz, 12-core, and 128-GB installed memory at ambient temperature. The optimum dimensions of the microchannel heat sink were investigated under a constant inlet volumetric flow rate (200 ml/h) and constant heat flux value (5 suns). The simulation stage was carried out using design of experiment (DoE) tool; custom and sampling type. This is connected to a response surface generic aggregation method (RSM). The optimization tools used a multi-objective genetic algorithm (MOGA) to determine the optimal candidate header lengths and channel height. At the DoE stage, 65 design points were generated for microchannel height and 31 points for header length.

#### Boundary conditions

**Figure 2:**
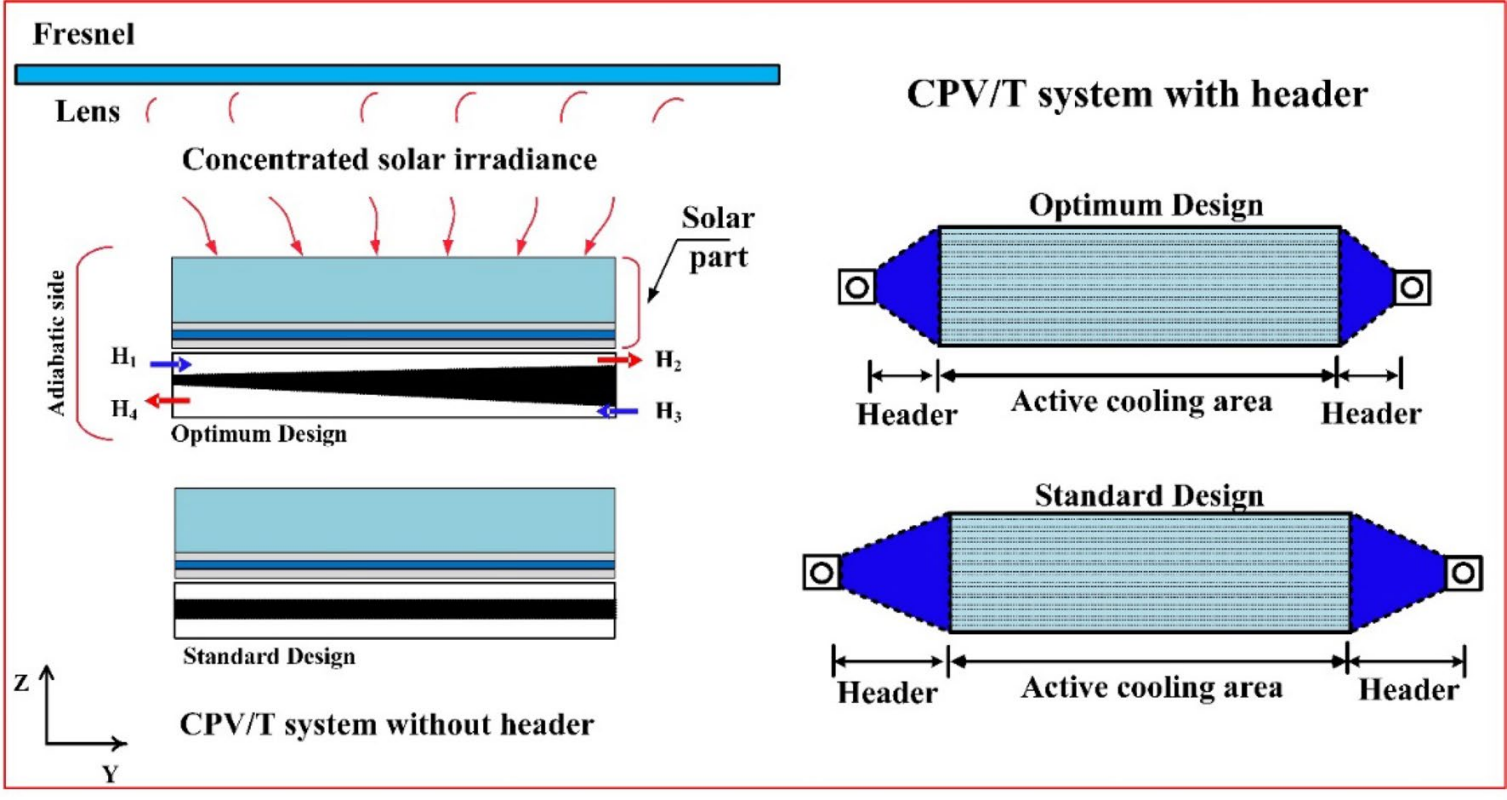
Two-dimensional boundary conditions for the DL-MCHS design where H1 and H2 are inlet and outlet top heights, H3 and H4 are inlet and outlet bottom heights.

Two numerical analysis were used in the current study. First, a two-dimensional optimization was implemented to optimize the channel height of the heat sink. Therefore, as seen in Fig. 2, the boundary conditions for this part considered water as a coolant medium that flows through channels at a rate of 200 ml/h and 5 suns concentration ratio. All surfaces have a no-slip boundary condition applied to them, with the sides being treated as adiabatic and the outlets were zero gauge pressure.

**Figure 3:**
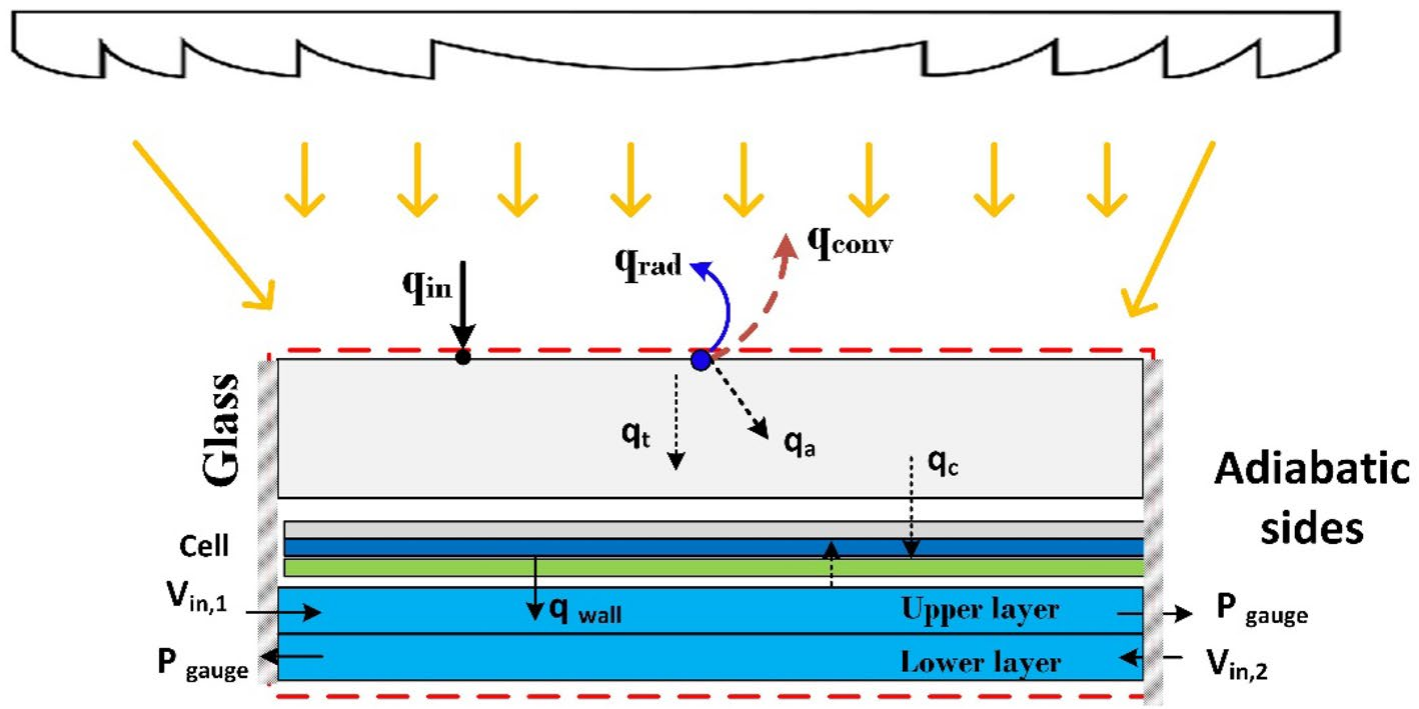
Three-dimensional boundary conditions for the CPV/T system.

The second part simulates the CPV/T system as a case study to calculate the overall system performance. The top surface is subjected to a mixture of heat transfer factors: convection and radiation^17^. With an ambient temperature of 25 °C and a glass emissivity of 0.85, the convective heat transfer coefficient was considered to be 9.89 W/m^2^ K. It was determined that both inflow ports had uniform coolant velocities inside the MCHS layers. The atmospheric pressure of the zero gauges was also specified in the outlet ports. For the DL-MCHS to operate with the optimum level of thermal efficiency, the rear surface was also assumed to be adiabatic, as shown in Fig. 3.

#### Governing equations

This section is divided into two main points. The first explains the governing equation for the coolant domain, while the second for the PV panel.

ANSYS Fluent module software was implemented for the coolant domain to solve the microchannel governing equations. This part contains the fluid domain, where the coolant flows through the mico-size channel for thermal management. The governing equations for the fluid domain, continuity, momentum and mass conservation equations are as follows^18^:

Mass conversation equation:

1 $$\nabla \cdot \left(\rho \overrightarrow{ V}\right)=0$$

Momentum equation:

2 $$\overrightarrow{V}\cdot \nabla \left(\rho \overrightarrow{ V}\right)=-\nabla P+\nabla \cdot \left(\mu \nabla \overrightarrow{V}\right)$$

Fluid energy equation:

3 $$\overrightarrow{V}\cdot \nabla \left(\rho {C}_{f} T\right)=\nabla \cdot \left(k \nabla T\right)$$

where $\rho$ and $\mu$ are coolant density and viscosity. $\overrightarrow{V}$ is the velocity vector and $P$ is the coolant pressure, respectively.

For the solid domain, the energy equation can be written as follows:

4 $$\nabla \left({\mathrm{k}}_{\mathrm{s}}.\nabla {\mathrm{T}}_{\mathrm{s}}\right)=0$$

For the PV module layers, the heat generated through the glass, the EVA, and the silicon layers can be calculated using the following equation^19^:

4 $${q}_{Ge}=\frac{\left(1-{\eta }_{cell}\right) \cdot G \cdot {\alpha }_{Ge} \cdot A}{V}$$

where, ${q}_{Ge}$ depend upon the overall concentrated solar radiation ($G$). This step is accomplished using the iteration technique until convergence. The following items are (${\alpha }_{Ge}$) is the absorptivity of the silicon cell^17^, ${\eta }_{cell}$ is the cell’s electrical efficiency, $A$ is ithe area of the top surface, and $V$ is the volume of the cell. The

fficiency ${\eta }_{cell}$ can be calculated by the following equation ^20^.

5 $${\eta }_{cell}={\eta }_{Ref}-\left[{\beta }_{Th} \left({T}_{cell}-{T}_{Ref}\right)\right]$$

where ${\eta }_{Ref}$ = 18% when the reference temperature ${T}_{Ref}$= 25 °C and CR = 5 Suns, and ${\beta }_{Th}$ is the thermal coefficient equal to 0.0045.

#### CPV/T simulation procedure

Following the selection of the optimal microchannel heat sink design, this part examines the simulation techniques for calculating the total performance of solar panels. The solution phases start with developing a complete model using modeler design software. Then, mesh independence tests were conducted to increase the accuracy and minimize the solution time. Finally, the Fluent modeler software was updated to include the operational condition. The boundary conditions for the current numerical investigation in Fluent were implemented using equations 1–8 at reference^14^. A source term is added to the glass cover, top EVA, and silicon layers based on equations 2–4^14^. The ongoing numerical solution is repeated until it reaches a temperature difference of 0.1 °C between two successive silicon wafer temperatures. The electrical efficiency is then updated, and the data for the other parameters are evaluated. For each flow rate, the above processes were repeated.

### Grid test and model validation

#### Grid test

**Figure 4:**
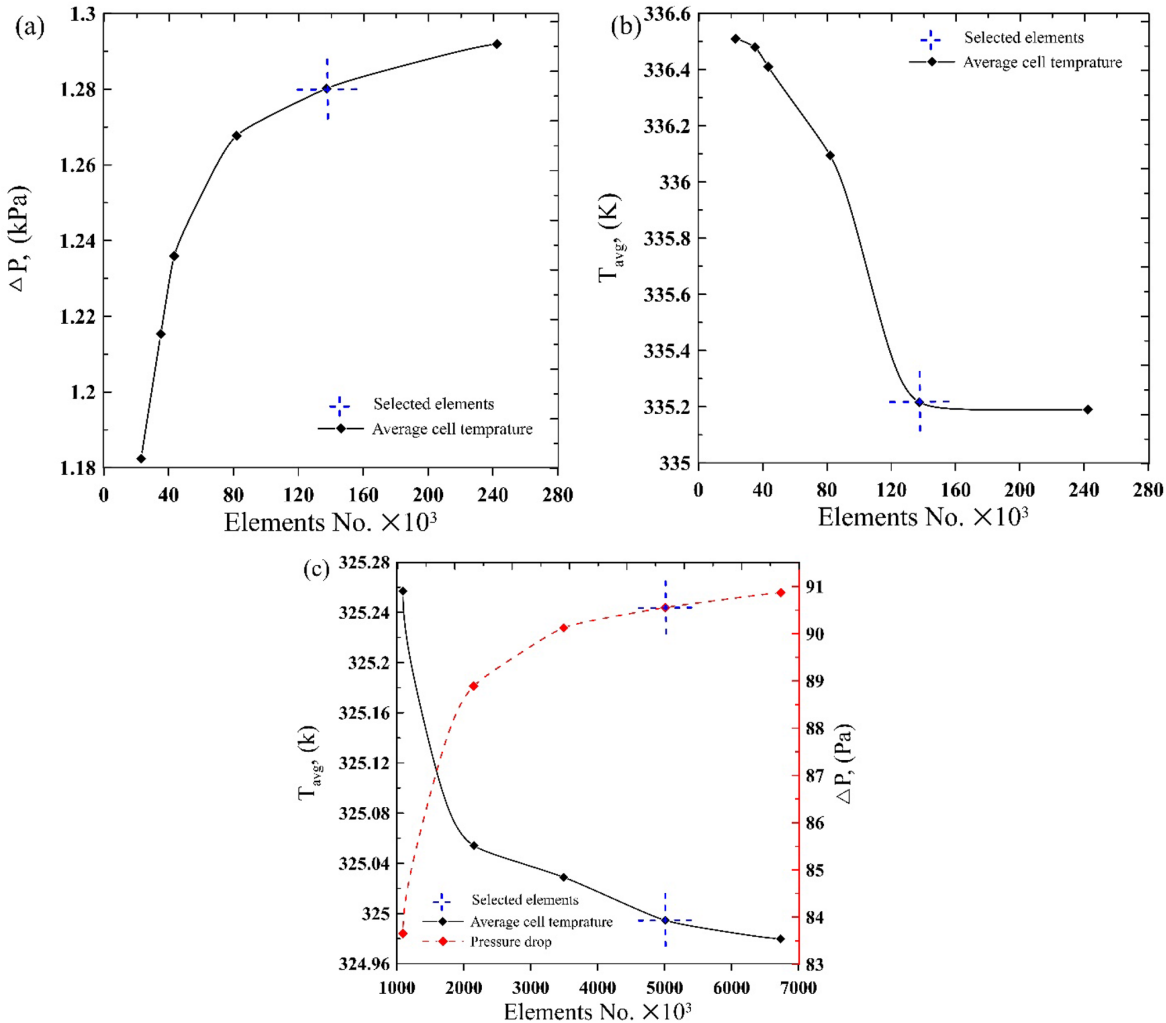
Mesh independence test for (**a**, **b**) 2D divergent microchannel without header, (**c**) CPV/T system.

The optimum number of elements was chosen when the surface pressure drop and average cell temperature stayed nearly unchanged. Figure 4 explains the full examination points of the current mesh sensitivity test for both models, the 2D and the 3D. Based on Fig. 4a and b, the grid of total mesh number 137448 was chosen for the divergent heat sink module. While 5010188 elements for the 3D CPV model as in Fig. 4c.

#### Model validation

**Figure 5:**
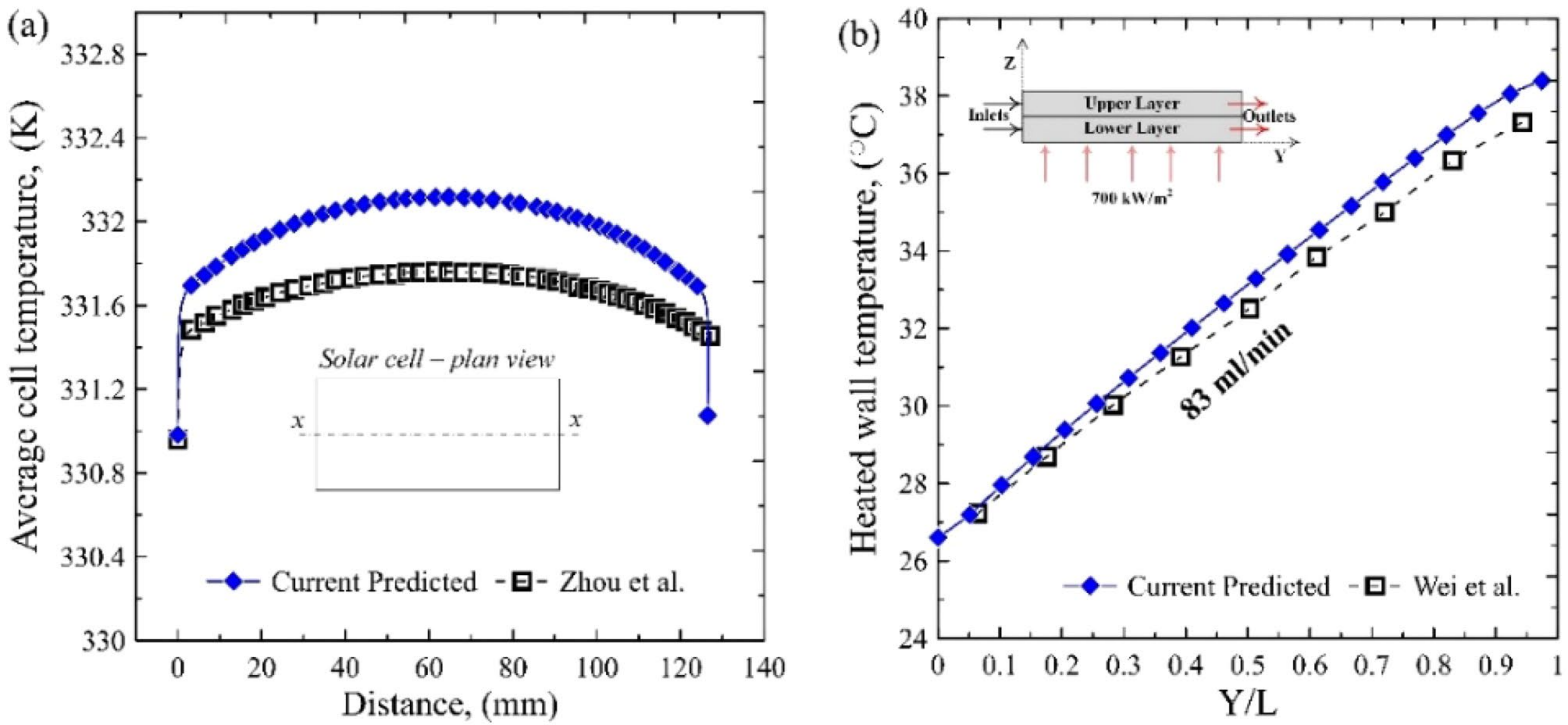
Model validation with (**a**) Zhou et al.^17^, (**b**) Wei et al.^21^.

By varying the amount of solar irradiance and ambient temperature, a careful validation process takes place to validate the current solar panel module with the available numerical results. As shown in Fig. 5a, there is a reasonable agreement between the predicted results of the current work and the numerical results of Zhou et al.^17^, with a maximum relative error of 4.4%. Wei et al.^21^ experimentally fabricated a microchannel heat sink for cooling electronic chips. Therefore, the heat sink model was validated with the experimental results of Wei et al.^21^. At 83 ml/min, the predicted results indicate a good agreement with a maximum deviation of 2.4%, as demonstrated in Fig. 5b.

## Results and discussion

### Optimization of the divergent microchannel

**Figure 6:**
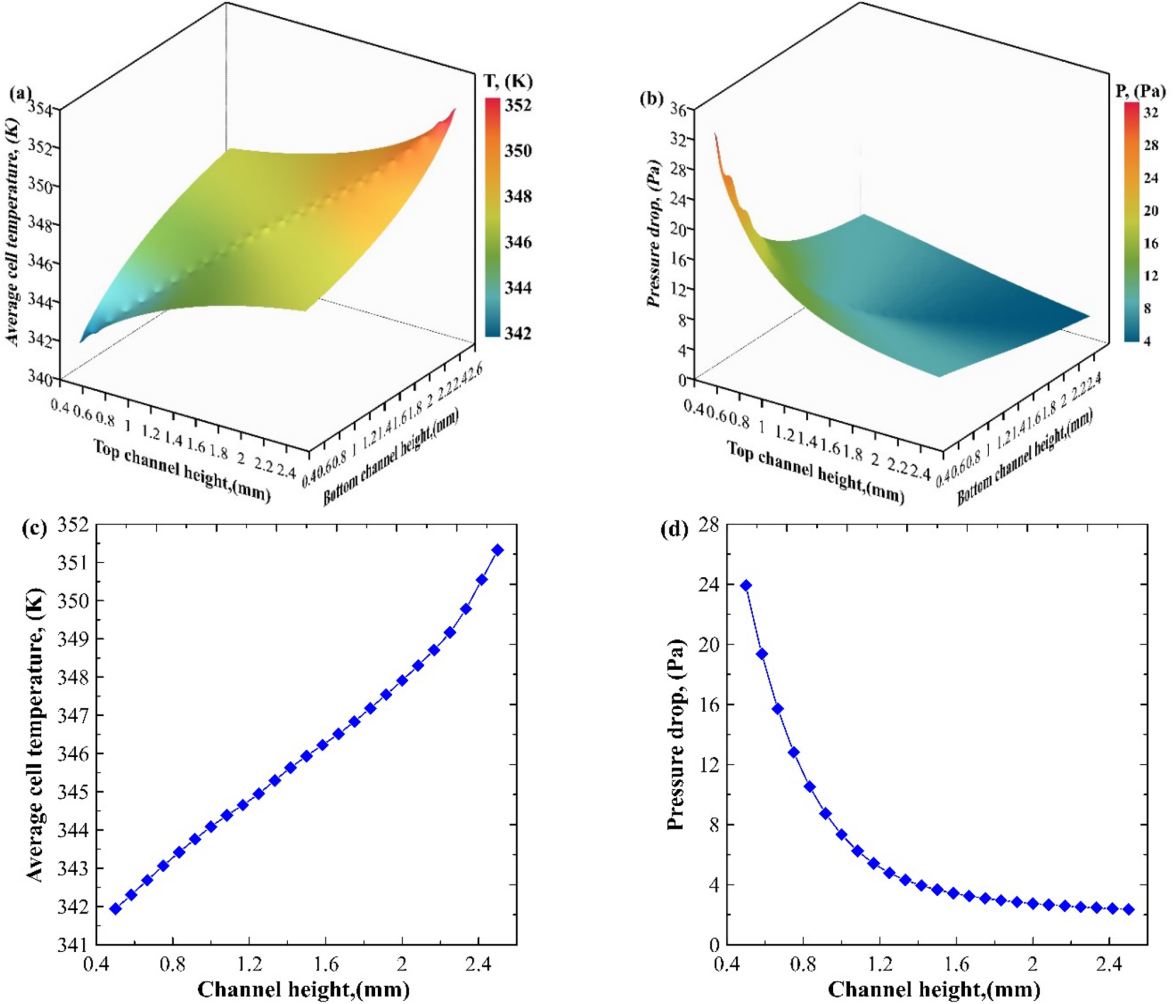
Surface response for temperature and pressure.

Figure 6 examines the surface response results between two important factors, average module temperature and pressure drop. The optimum channel heights (Hch) value was evaluated using a multi-objective genetic algorithm solution type. As shown in Fig. 6a, the reported average module temperature was significantly increased on the bottom side with increasing the values of channel height, while the top reported a slight increase. Similarly, there is a slight pressure increase with decreasing the channel height at the bottom layer, as illustrated in Fig. 6b. However, the upper reported a noticeable increase. Pressure drop and temperature responses were important steps to show the best conditions for optimizing the divergent microchannel (H_ch_). A balance for achieving lower temperature and pressure values should be the key factor for optimizing the channel height. Therefore, as shown in Fig. 6c and d, the best channel height was selected regarding minimum pressure drop and minimum wall temperature; the optimum height (H_ch_) is 0.5 mm, as depicted in Fig. 6c and d.

### Optimization length of headers

**Figure 7:**
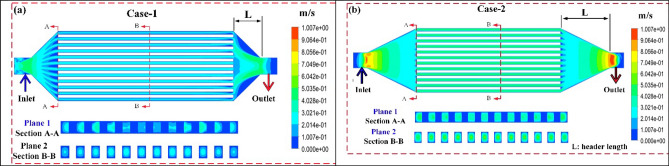
DL-MCHS header optimization.

The selected design point of the divergent channels (H_ch_) was used as input data for header optimization. A 31 design points were numerically solved using 3D solving software. The most important factor for optimizing the length of headers is achieving a uniform flow through channels without any turbulence. Based on the current literature survey, it is recommended to elongate the header length to gain uniform flow over channels and prevent heat sink from maldistribution. As shown in Fig. 7a and b, the velocity ratio of coolant was evaluated in a line located in the middle of the heat sink; the line is perpendicular to the flow stream. The best design point is expected to attain a unity velocity ratio through each channel. Therefore, the best and worst design points are demonstrated in Fig. 7. A non-uniform distribution of coolant over channels is generated at a lower velocity ratio. Thus, various drawbacks affected this design (case 1), including temperature nonuniformity, hot-spot, and physical deformation. For further clarification, case 1 reported higher recirculation values at the inlet with a higher velocity ratio. However, case 2 achieved a more uniform flow distribution with a lower recirculation area at inlets.

### System performance assessment

The major purpose of the current work is to optimize a new cooling mechanism to achieve temperature homogeneity through the PV panel. Therefore, the selected dimension was employed in a 3D numerical investigation to evaluate the overall system performance for the new structure of polycrystalline solar panel suggested by the authors’ earlier work^14^. 

**Figure 8:**
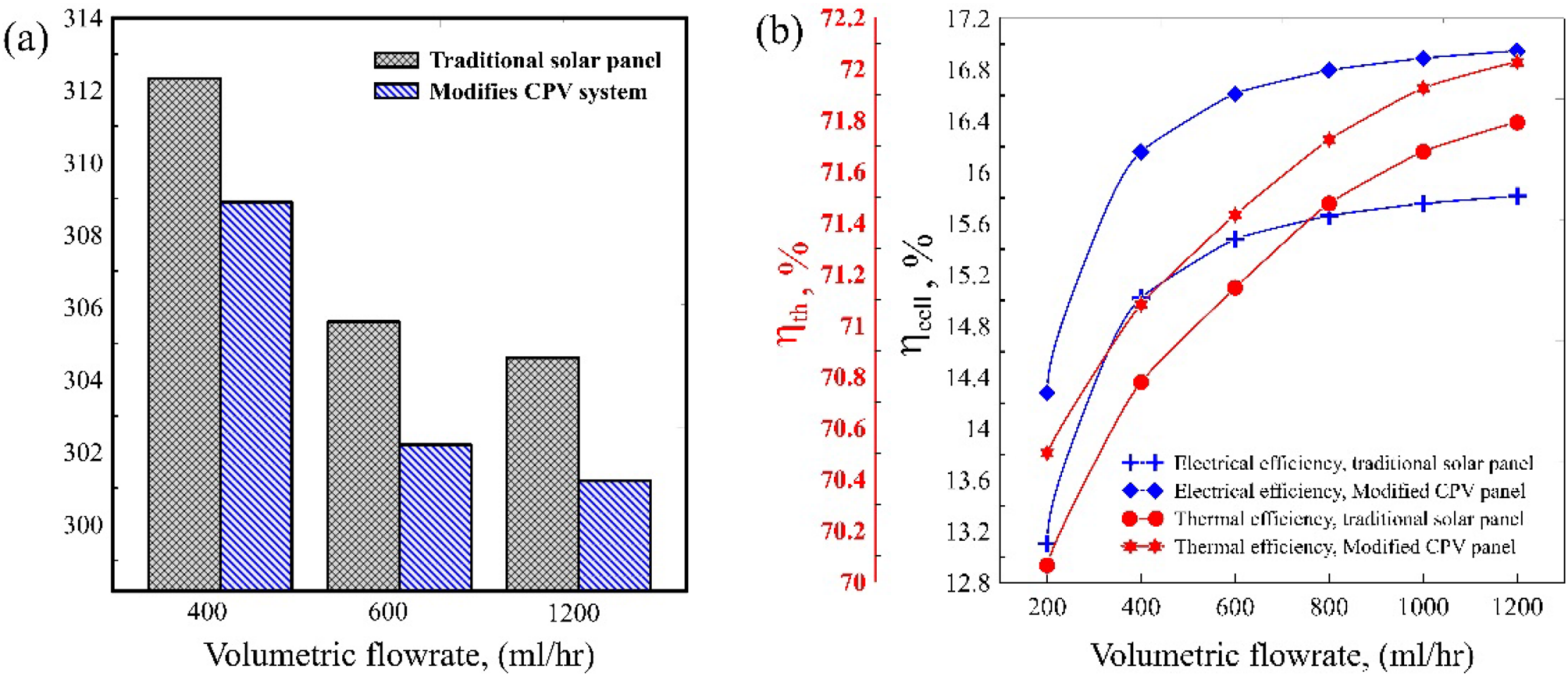
Electrical and thermal efficiencies for the traditional and modified solar panel at 5 suns concentration ratio.

Average cell temperature is the most important parameter for calculating the CPV system's performance^22^. In addition, the flow direction of coolant inside the heat sink plays a significant role in achieving temperature uniformity over the cell surface; counter flow (CF) operation is the best flow condition for achieving such uniformity^13^. Therefore, the optimized heat sink operated under a CF and 5 suns concentration ratio. Figure 8a explains the variation of average cell temperature with flow rate compared with a traditional solar cell^15^. With increasing coolant flow rate, the reported average cell temperature decreased gradually. This is due to water movement inside microchannels rejecting most of the generated thermal energies over the cell; the reported average cell temperature reduced from 312.15 to 301.2 k. Besides, the flow stream of coolant in the upper layer of the microchannel heat sink cooled with the opposite coolant flow in the lower layer. Generally, solar cells suffer from generating hot spots as the concentrated solar radiation will focus on one point^23^. Therefore, cooling photovoltaic modules prevent panels from the overwhelming heat. Cell electrical (η_el_) and thermal (η_th_) efficiencies are considered significant factors when evaluating total solar panel performance. Compared with traditional solar panels, the electrical and thermal efficiencies increased to 17.45% and 69.6%, respectively, instead of 15.8% and 61.3%, as shown in Fig. 8b^22^.

## Conclusion

Two optimization methods have been employed to optimize the microchannel heat sink geometry. The first study is conducted to optimize the height of the microchannel channel under a constant width value. While the second is employed for evaluating the best length of headers to prevent the water from recirculations inside the heat sink. The results reported a great balance for achieving more uniformity of coolant inside the microchannel and inlet/outlet headers. The best condition was reported for a channel height of 0.5 mm and header length of 20 mm. The selected design points were implemented in a complete three-dimensional model for evaluating the performance of a solar/thermal module. The electrical efficiency improved when the coolant flow rate increased; the greatest reported electrical efficiency was 17.45%. The capital cost of installing the experimental unit may rise due to the manufacture of the existing heat sink design. However, this system can increase electrical yield, operate within the recommended condition, and use the rejected heat for other domestic purposes.

## Data Availability

The data produced during this study are included in this published article and all relevant data are available from the corresponding author at reasonable request.
